# Detection of Insect-Specific Flaviviruses in Mosquitoes (Diptera: *Culicidae*) in Northeastern Regions of South Africa

**DOI:** 10.3390/v13112148

**Published:** 2021-10-25

**Authors:** Milehna M. Guarido, Kamini Govender, Megan A. Riddin, Maarten Schrama, Erin E. Gorsich, Basil D. Brooke, Antonio Paulo Gouveia Almeida, Marietjie Venter

**Affiliations:** 1 Zoonotic Arbo- and Respiratory Virus Program, Centre for Viral Zoonoses, Faculty of Health Sciences, University of Pretoria, Pretoria 0031, South Africa; milehna@gmail.com (M.M.G.); u16037759@tuks.co.za (K.G.); PAlmeida@ihmt.unl.pt (A.P.G.A.); 2 UP Institute for Sustainable Malaria Control (UP ISMC), Faculty of Health Sciences, University of Pretoria, Pretoria 0007, South Africa; megan.riddin@up.ac.za; 3 Institute of Environmental Sciences, Leiden University, 2333 CC Leiden, The Netherlands; maartenschrama@gmail.com; 4 School of Life Sciences, University of Warwick, Coventry CV4 7AL, UK; eringorsich@gmail.com; 5 The Zeeman Institute for Systems Biology & Infectious Disease Epidemiology Research, University of Warwick, Coventry CV4 7AL, UK; 6 Centre for Emerging Zoonotic & Parasitic Diseases, National Institute for Communicable Diseases/NHLS, Johannesburg 2192, South Africa; basilb@nicd.ac.za; 7 Wits Research Institute for Malaria, School of Pathology, University of the Witwatersrand, Johannesburg 2000, South Africa; 8 Institute of Hygiene and Tropical Medicine (IHMTNOVA), Medical Parasitology Unit/GHTM, NOVA University of Lisbon, 1349-008 Lisboa, Portugal

**Keywords:** flavivirus, insect-specific, cell-fusing agent virus, mosquitoes, *Aedes*, *Anopheles*, *Culex*, Africa region

## Abstract

Mosquitoes in the *Aedes* and *Culex* genera are considered the main vectors of pathogenic flaviviruses worldwide. Entomological surveillance using universal flavivirus sets of primers in mosquitoes can detect not only pathogenic viruses but also insect-specific ones. It is hypothesized that insect-specific flaviviruses, which naturally infect these mosquitoes, may influence their vector competence for zoonotic arboviruses. Here, entomological surveillance was performed between January 2014 and May 2018 in five different provinces in the northeastern parts of South Africa, with the aim of identifying circulating flaviviruses. Mosquitoes were sampled using different carbon dioxide trap types. Overall, 64,603 adult mosquitoes were collected, which were screened by RT-PCR and sequencing. In total, 17 pools were found positive for insect-specific *Flaviviruses* in the mosquito genera *Aedes* (12/17, 70.59%) and *Anopheles* (5/17, 29.41%). No insect-specific viruses were detected in *Culex* species. Cell-fusing agent viruses were detected in *Aedes aegypti* and *Aedes caballus*. A range of anopheline mosquitoes, including *Anopheles coustani*, *An. squamosus* and *An. maculipalpis*, were positive for *Culex* flavivirus-like and *Anopheles* flaviviruses. These results confirm the presence of insect-specific flaviviruses in mosquito populations in South Africa, expands their geographical range and indicates potential mosquito species as vector species.

## 1. Introduction

Members of the genus *Flavivirus* are emerging arthropod-borne viruses that have a great impact on global health. Important mosquito-borne viruses belonging to this genus include West Nile, yellow fever, dengue and Zika viruses. In areas where these viruses occur, it is important to carry out mosquito surveillance as a tool to understand the dynamics of arboviral circulation. One strategy to quantify arboviral circulation is to use RT-PCR, more specifically RT-PCR with consensus primers, followed by sequencing to identify arboviruses at the species and genotype level [1]. While providing in-depth information on circulation of known arboviruses, this strategy has also enabled researchers to identify previously unknown viruses, including apparent insect-specific flaviviruses (ISFV).

Insect-specific viruses (ISV) naturally infect insects and do not appear to replicate in vertebrate cells or affect vertebrates [2]. Because they are present in both male and female mosquitoes, vertical transmission has been suggested as a mechanism for their maintenance in nature [3]. The importance of this group has been neglected due to the fact they do not infect vertebrate cells; however, this changed with the discovery of some ISFVs, which enhance or suppress the replication of medically important flaviviruses in co-infected mosquito cells [4,5,6], influencing their vector competence for zoonotic arboviruses. The first described virus restricted to an insect host was a *Flavivirus* member, a virus named cell-fusing agent virus (CFAV) from an *Aedes aegypti* cell line, which produced massive syncytia in *Aedes albopictus* cells (C6/36) but was unable to replicate in vertebrate cell lines [7]. Since then, novel insect-specific viruses have been reported in wild-caught mosquitoes in different mosquito genera and in different parts of the world. The ISFVs have distinguishing features. Insect-specific flaviviruses can produce DNA forms of their genomic RNA [8], and integrated sequences related to ISFVs can be present in the genome of certain species of mosquitoes [8,9]. This feature can be detected for other members of the Flavivirus genus but also in members of insect-specific Rhabdoviruses and Phenuiviruses [10,11,12]. Phylogenetic analyses of the ISFVs have shown distinct lineages in the genus *Flavivirus*, with lineages roughly grouped according to their host mosquito species [13]. There are known two clades of ISFVs named “classical ISFVs” (cISFV) and “dual-host-affiliated ISFVs (dISFV)” [4] The first is the most diverse and presents ubiquitous host and geographical distribution, among them are *Aedes* flaviviruses, CFAVs, Kamiti river virus and *Culex* flaviviruses. However, dISFVs, such as Lammi virus and Chaoyang virus, are considered phylogenetically related to arboviruses [14].

In southern Africa, little is known about this group of viruses but a range of zoonotic flaviviruses occurs in these areas such as the West Nile and Wesselsbron viruses, making them potentially relevant for understanding their dynamics. Therefore, this study was part of a larger entomological surveillance program using a broad arboviral screening methodology with the aim of identifying arbovirus circulation among *Culicidae* species collected in different habitats across five provinces of South Africa. In this study, we discussed only the variety of insect-specific flaviviruses detected in that entomological surveillance program.

## 2. Materials and Methods

### 2.1. Study Area and Mosquito Collection

Entomological surveillance was conducted in the northeastern parts of South Africa in sentinel and ad hoc sites located in five different administrative provinces in South Africa, namely Gauteng, Limpopo, North West, Mpumalanga and KwaZulu-Natal Provinces. The sites were established in four different habitat types: urban, peri-urban, rural and conservation areas. The survey occurred from January 2014 to May 2018. Trapping was carried out from 15:30–16:00 to 5:00–8:00, and sampling was conducted for 1 to 3 consecutive nights per site using multiple types of carbon dioxide (CO_2_)-baited traps: mosquito net, CDC miniature light (BioQuip Products, Rancho Dominguez, CA, United States), and BG-Sentinel (BioGents Corporation, Regensbourg, Germany). Mosquitoes were collected from the net traps using hand-held aspirators and transferred to mesh-topped polystyrene cups. CDC miniature light traps were hung at least 1.5 m from the ground, baited with CO_2_ and multiple lights of differing wavelengths (incandescent, LED, UV white and UV black). BG-Sentinel traps were additionally baited with a non-toxic lure. Traps were placed at least 80 m apart to ensure no interference occurred. Collected mosquitoes were immediately euthanized by freezing and were morphologically identified to species level using published keys and descriptions [15,16,17,18]. Females were pooled (predominantly ≤50 individuals per pool) by species, site, and month of collection, and preserved at −80 °C until testing. Collections from January to June were selected for screening. 

### 2.2. Mosquito Processing and RT-PCR

Mosquito pools were homogenized, and viral RNA extracted in a biosafety level 3 laboratory at the University of Pretoria, Centre for Viral Zoonoses. Briefly, five sterile glass beads were placed in microcentrifuge tubes (Eppendorf, Germany) containing 2000 µL reconstituted minimum essential medium (MEM) (Life Technologies) and shaken vigorously in a TissueLyser. The homogenates were clarified by centrifugation at 3000× *g* for 30 min at 4 °C and stored at −80 °C. Viral RNA was extracted from 200 µL homogenate using the RNeasy^®^ mini kit (Qiagen, Valencia, CA, USA) according to the manufacturer’s instructions for purification of total RNA from animal tissues. This protocol includes a gDNA eliminator column which removes all genomic DNA present in the homogenate, according to the manufacturer. 

Extracted viral RNA was screened for viruses in the *Flavivirus* genus by targeting fragments of the viral non-structural protein 5 (NS5) gene using a published genus-specific nested real-time PCR with WNV-specific probes as described in Zaayman et al. 2009 [19]. In brief, a first round RT-PCR were performed using genus specific primers FU1 [20] and 9317 [19] using Superscript III One-Step RT-PCR System with Platinum Taq DNA polymerase (Thermo Fisher Scientific, MA, USA): 5× RT-PCR Reaction Buffer, RT-PCR Superscript Enzyme Mix in combination with 20 pmol of each primer were mixed with nuclease free water to a total volume of 50 µL reaction following addition of 10 µL extracted RNA. The reaction mix was subjected to initial incubation of 50 °C for 30 s, 94 °C for 7 min, followed by 35 PCR cycles: 94 °C for 10 s, 55 °C for 30 s and 68 °C for 1 min and final extension for 68 °C for 5 min. 

This was followed by a nested real-time PCR with genus-specific primers (FS778 and CFD2) with the West Nile virus hydrolysis probes (WNV) 1 and 2 [19] using the Hybprobe assay (Roche Life Science, Mannheim, Germany) on the Roche LightCycler^®^ 2.0 resulting in a fragment of 180 bp PCR product: 10 pmol of each primer FS778 and CFD2, 10 pmol of the WNV specific HybProbes (10 pmol) is combined with the LightCycler^®^ FastStart DNA MasterPLUS HybProbe (Roche Life Science, Mannheim, Germany) master mix. Cycling conditions were as follows: 95 °C for 10 min, 45 cycles of 95 °C for 10 s, 52 °C for 1 min and 72 °C for 1 min, followed by a melting curve at 95 °C for 0 s, 30 °C for 30 s and 80° C for 0 s (continuous) [19].

This study was part of a larger entomological surveillance program. The use of genus-specific primers allowed for the identification of all flavivirus positives, while WNV-specific probes rapidly identified WNV-positive pools. West Nile virus data will be reported elsewhere. 

In samples that tested positive, an additional heminested PCR was performed using MAMD and Flavi-2 published primers followed by a second round using Flavi-1 and Flavi-2 primers to detect an 850 bp fragment of the same gene [1]. Briefly, each reaction contained 10 μL of RNA, 1 μL of each primer MAMD and Flavi-2, 25 μL of 2X Superscript III reaction, 2 μL Superscript III Reverse Transcriptase, 11 μL of nuclease free water in a reaction volume of 50 μL reaction. Cycling conditions were as follows: 50 °C for 30 min, followed by 94 °C for 15 min, 45 cycles of 94 °C for 45 s, 50 °C for 45 s and 72 °C for 1 min and one step of 72 °C for 10 min. The second round was carried out using the Platinum Taq DNA Polymerase system. Each reaction contained 2 μL of the first-round product, 1 μL of each primer Flavi-1 and Flavi-2, 5 μL of 10X PCR buffer, 2 μL 50 mM MgCl2, 1 μL 10 mM dNTPS, 37.8 μL nuclease free water and 0.2 μL Platinum Taq DNA Polymerase. Cycling condition were as follows: 94 °C for 15 min, 45 cycles of 94 °C for 1 min, 55 °C for 1 min, 72 °C for 90 s and one step of 72 °C for 10 min. 

All the primers used in the flavivirus detections are shown at Table S1.

### 2.3. Molecular Identification of the Mosquito Positive Pools

To confirm the morphological identification of the positive pools to species level where possible, DNA was extracted from 50 µL of each homogenate using the DNeasy Blood and Tissue Kit (Qiagen, Valencia, CA, USA), according to the manufacturer’s instructions. The DNA barcode region of mtDNA of the subunit I of the cytochrome oxidase (COI) gene was amplified using universal primers [21]. The 50 μL PCR reaction consisted of 5 μL of the extracted DNA, 1 μL of 10 mM dNTPs, 10 μL of buffer, 0.5 μL Phusion High Fidelity DNA Polymerase (ThermoFisher Scientific, Waltham, MA, USA) and 1 μL of 20 μM of each primer. PCR reaction conditions were as follows: 98 °C for 30 s followed by 35 cycles of 98 °C for 30 s, 52 °C for 45 s and 72 °C for 30 s, with a final extension of 72 °C for 5 min. 

### 2.4. Gel Electrophoresis and Sequencing

All products of the expected size were viewed on a 2.0% agarose gel containing ethidium bromide. Amplicons of the correct size were excised from the gel and purified using a Zymoclean Gel DNA Recovery Kit (Zymo Research, Irvine, CA, USA) according to the manufacturer’s instructions. Purified amplicons were bidirectionally sequenced using the BigDye Direct Sanger Sequencing Kit (ThermoFisher Scientific, Waltham, MA, USA) and sent to the University of Pretoria DNA sequencing facility or Inqaba Biotec (Pretoria, South Africa) for Sanger sequencing.

### 2.5. Data Analyses

The sequences produced were analyzed in CLC Main workbench version 8.0.1 [22] and were compared with the databases available in the NCBI GenBank [23] dataset using the BLAST program. The sequences from this study were deposited in NCBI GenBank, and the small fragments of the 180 bp region of NS5 are available in the supplementary material. Sequences were trimmed to remove the primer sequences. Reference sequences for phylogenetic comparison were downloaded from GenBank [24]. Sequences included previously reported sequences of CFAV and mosquito flaviviruses and representative sequences of the genus *Flavivirus*. For COI, representative mosquito sequences were selected on GenBank [24] and BOLD [23] databases. Multiple sequence alignments were compiled using the online version of MAFFT (Multiple Alignment using Fast Fourier Transform) with default parameters (https://mafft.cbrc.jp/alignment/software/, accessed on 2 December 2020) [25]. Best model fit was tested, the p-distance matrix (p-distance is the proportion of nucleotide sites at which two sequences being compared are different) and the maximum likelihood tree was built in MEGA 7 [26].

## 3. Results

### 3.1. RT-PCR Screening 

Overall, 64,603 adult mosquitoes were collected at the sentinel and ad hoc sites [27]. The most abundant genus collected was *Culex* (38.90%, N = 25,131) followed by *Anopheles* (33.27%, N = 21,494), *Aedes* (18.63%, N = 12,037), *Mansonia* (6.17%, N = 3987) and other genera combined (3.03%, N = 1954, *Uranotaenia*, *Aedeomyia*, *Ficalbia*, *Coquillettidia*, *Mimomyia*, *Culiseta* and *Eretmapodites*) [27].

**Figure 1:**
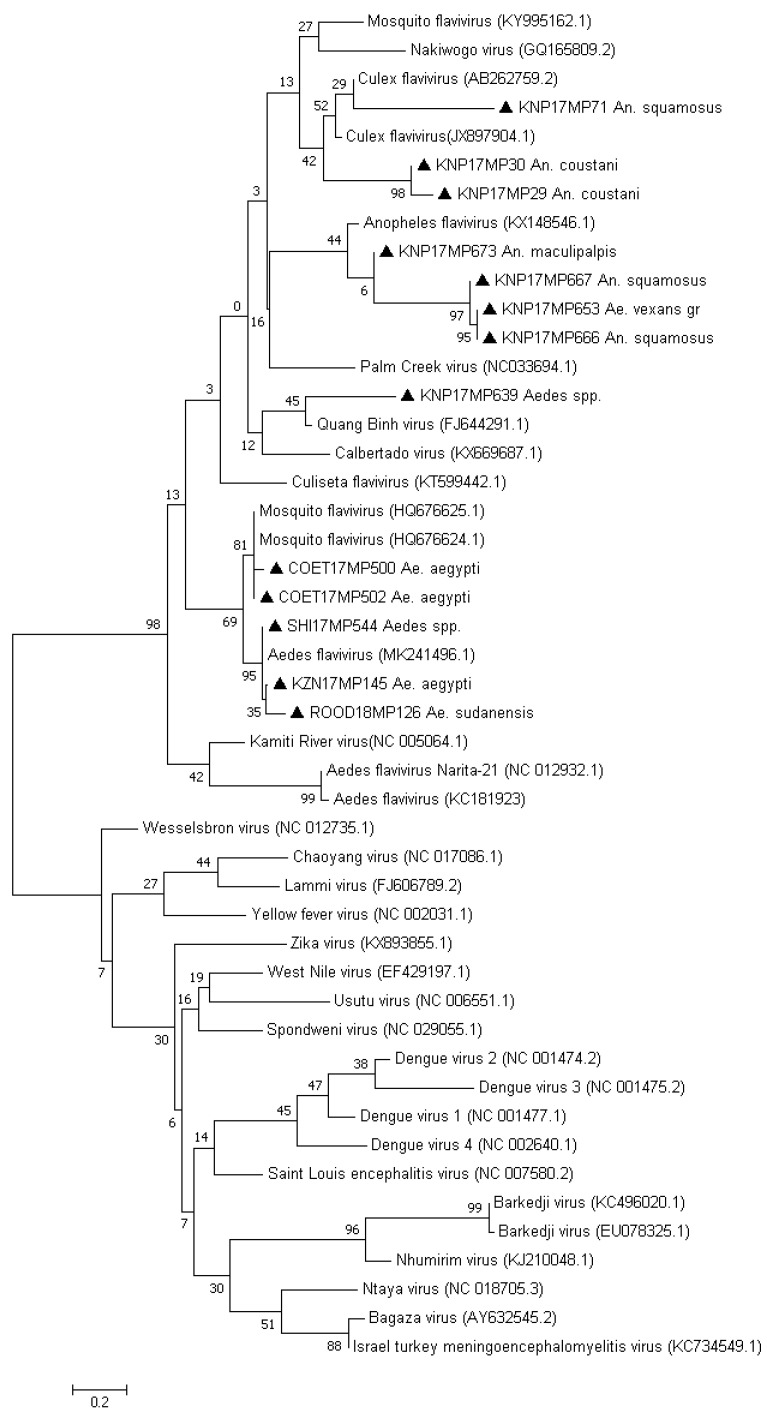
Phylogenetic tree of the mosquito-specific *Flavivirus*-positive pool sequences based on the 47 sequences and 180 bp of the NS5 gene. The tree was constructed by employing the program MEGA 7, using the maximum likelihood method based on the Kimura2-parameter model and 1000 bootstrap replicates. The tree with the highest log likelihood (−3048.61) is shown. GenBank accession numbers are indicated. Numbers on internal branches indicate bootstrap values. Samples that are part of this study are marked with a triangle.

During the study period, only mosquitoes collected from January to June were screened for the *Flavivirus* genus. A total of 39,035 mosquitoes were grouped into 1462 pools. Of these, a total of 17 pools tested positive by RTPCR and were identified as ISFVs following sequencing, BLAST searches and maximum likelihood phylogenetic analysis of the 180 bp flavivirus-positive NS5 PCR fragment (Table 1 and Table S2, Figure 1). Clustering with the ISFV’s was supported with a bootstrap value of 99%, although none of the individual viruses clustered with published ISFVs with significant bootstrap statistics on sub-branches. Eleven of these were in *Aedes* mosquitoes (11/17, 64.7%), while the remaining six were from *Anopheles* mosquitoes (6/17, 35.3%).

**Figure 2:**
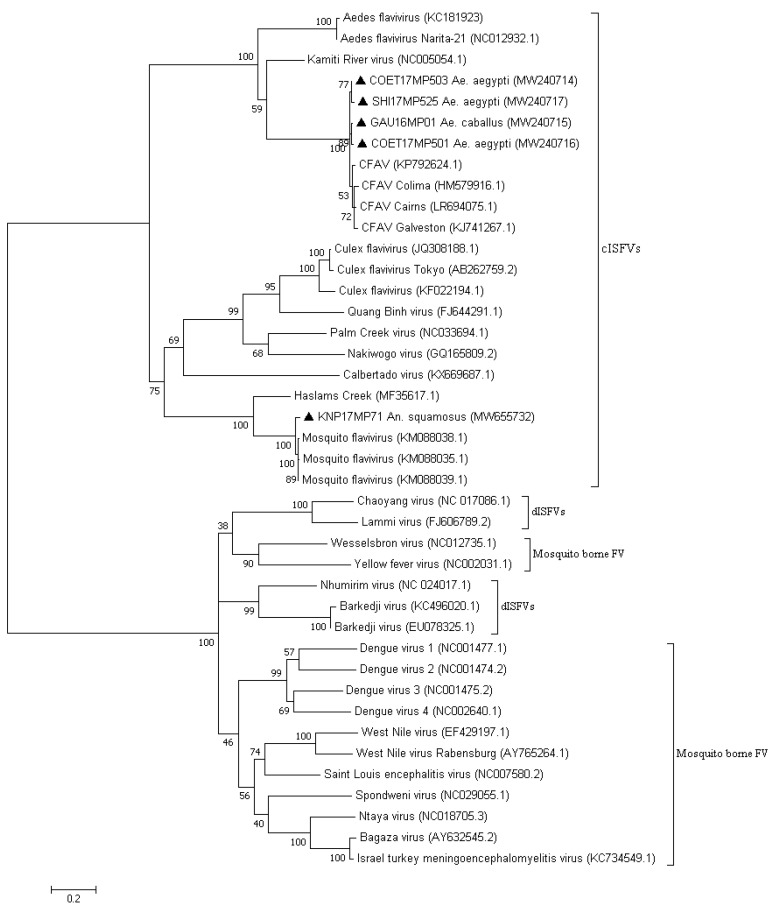
Phylogenetic tree of the mosquito Flavivirus-positive sequences based on the 41 sequences and 850 bp of the NS5 gene. The tree was constructed by employing the program MEGA 7, using the maximum likelihood method based on the Tamura-3 parameter model and 1000 bootstrap replicates. The tree with the highest log likelihood (−16,417.80) is shown. GenBank accession numbers are indicated. Numbers on internal branches indicate bootstrap values. Samples that are part of this study are marked with a black triangle. cISFVs: classical insect-specific flaviviruses; dISFVs: dual host-related insect-specific flaviviruses; FV: flaviviruses.

Flavivirus RT-PCR-positive samples identified in *Aedes aegypti* and *Ae. caballus* were identified as CFAV by sequencing, followed by BLAST searches and phylogenetic analysis of the 180 bp PCR fragment (4/17, 23.53%) (results not shown). A larger area of the NS5 gene region could be amplified for all pools and sequenced. The maximum likelihood phylogeny based on the larger fragment (850 bp) confirmed that all samples clustered with previously published sequences of CFAV (Figure 2). P-distance analyses identified a high nucleotide similarity ranging from 93.54 to 98.03% (Table S3).

The sequences produced in this study clustered with the classic insect-specific mosquito flaviviruses (cISFV), which included sequences identified as mosquito flaviviruses and CFAV. Three pools of *Ae. aegypti* (3/17, 17.6%), two pools of *Aedes* spp. (2/17, 11.7%) and one for the *Ae. vexans* group (1/17, 5.8%) clustered together with published mosquito flaviviruses. One pool of *Ae. sudanensis* (1/17, 5.8%) clustered with Quang Binh virus, an insect-specific flavivirus previously isolated from *Culex* mosquitoes in China, although not with significant bootstrap support. 

Two *An. coustani* pools were designated as *Culex* flaviviruses-like (2/17, 17.6%). These two pools from the Kruger National Park (KNP) (KNP17MP29 and KNP17MP30) clustered with sequences of insect-specific *Culex* flaviviruses (CxFV), although not with significant bootstrap values, and were on a separate branch to the known *Culex* flaviviruses. Since the analyses were based on a small segment and these viruses were detected in *Anopheles* mosquitoes in this study, these samples were designated as CxFV-like. One pool of *An. maculipalpis* and three pools of *An. squamosus* clustered with mosquito flaviviruses, although on a separate branch than the *Aedes* mosquito flaviviruses. There were no ISFVs detected in *Culex* mosquitoes. 

Of the samples that clustered with the cISFV, only one sample collected in 2017 from the KNP (KNP17MP71) identified as *An. squamosus* pool could be amplified by the other generic genus-specific flavivirus PCR to obtain a larger 850 bp fragment of the NS5 gene for use in maximum likelihood phylogeny (Figure 2). The larger sequences confirmed their clustering with the mosquito flaviviruses (Figure 2). P-distance analyses identified nucleotide similarity ranging from 37.57 to 99.73% between the cISFVs (Table S4). Nucleotide similarities from the amplified sample collected (KNP17MP71) were higher between the published sequences of mosquito flavivirus from Kenya, and the values were 96.58% and 96.86% (Table S3). Limited conservation in the NS5 region between the cISFV may explain why the other cISFV-positive pools could not be amplified by the PCR targeting larger fragments. This sample was similar to the other mosquito flaviviruses identified in *An. squamosus* based on the 180 bp region, suggesting that these were similar species.

### 3.2. Molecular Identification of the Mosquito Positive Pools

**Figure 3:**
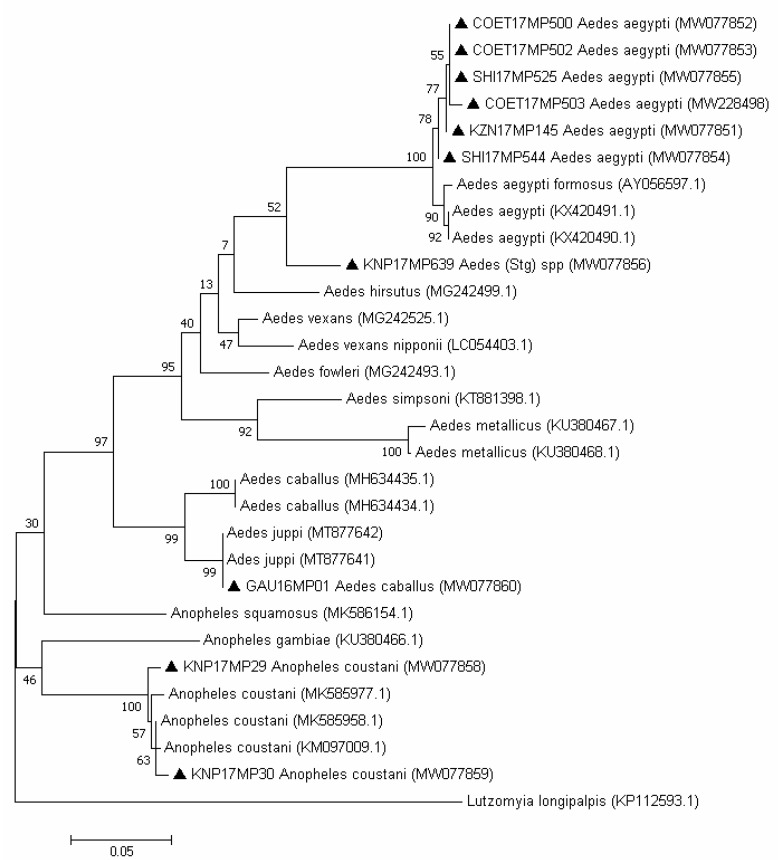
Phylogenetic tree of the mosquito pool sequences based on the 30 sequences and 507 bp of the COI gene. The tree was constructed by employing the program MEGA 7, using the maximum likelihood method based on the general time reversible model and 1000 bootstrap replicates. The tree with the highest log likelihood (−2554.03) is shown. GenBank accession numbers are indicated. Numbers on internal branches indicate bootstrap values. Samples that are part of this study are marked with a black triangle.

Most of the mosquito DNA extracted from the ISFV-positive pools could successfully be amplified using the COI gene PCR, and the morphological identification of the mosquitoes matched the phylogenetic analysis. Seven out of seventeen samples could not be amplified by PCR successfully, possibly because they were a mix of morphologically damaged species or due to degraded nucleic acid. The 507 bp COI region amplified from the mosquito pools was used to build a maximum likelihood tree for the remainder (Figure 3). The results show the evolutionary distances using the general time reversible model [28] with 1000 bootstrap replications [29]. Morphological identifications of *An. coustani* and *Ae. aegypti* were confirmed by COI segment analysis. A sample collected in Boschkop in 2016 (GAU16MP01), which was morphologically identified as *Ae. caballus*, showed a sequence identity of 96.45% with *Ae. caballus* from Iran (GenBank accession number MH634433.1), and clustered with sequences of *Ae. juppi* collected in South Africa.

## 4. Discussion

The use of generic *Flavivirus* genus primers revealed presence of ISFVs in pools of different species of mosquitoes collected over a four-year period across the northeastern provinces of South Africa. Based on sequencing and phylogenetic analysis of the 180 bp NS5 PCR product obtained through this screen, the positive pools were shown to cluster with the cISFVs (13 positive pools) and CFAV (4 positive pools). A larger region of NS5 could be amplified for all four of the latter and were confirmed to be CFAV, with 96% identity to known viruses. None of the positive pools that clustered with the cISFV had significant bootstrap values with specific isolates on GenBank, although several clustered with mosquito flaviviruses. Five positive pools identified in Aedes clustered closely with published mosquito flavivirus (HQ676625.1) but on a separate branch to those identified in *Anopheles*, while three *An. coustani*-positive pools clustered with Culex flaviviruses but also on a separate branch, suggesting a phylogenetic distinction according to the vector species. Finally, KNP17MP639 clustered with a Quang Binh virus (FJ644291.1) that was isolated from Culex mosquitoes in China. For most sequences of ISFVs detected in this study, we were unable to amplify larger fragments of the NS5 gene, except for one pool detected as mosquito flavivirus and four pools detected as CFAVs. As mentioned previously, insect-specific flaviviruses can produce DNA forms from their genomic RNA, and integrated sequences related to them can be present in genomes of some mosquito species [8,30] such as *Ae. aegypti* and *Ae. albopitus* [8,9]. To control for this, we used RNA extraction methods with genomic DNA eliminator columns that removed genomic DNA present in the mosquito homogenate; however, we could not ascertain, at this point, whether the small fragments that could not be confirmed by a larger PCR were DNA integrated in the host genome. However, the low level of conservation identified between the mosquito flavivirus in the 850 bp region suggested that these viruses might also not be detected by the primers that were designed to detect mammalian flaviviruses. The CFAVs that were highly conserved could all be amplified in the region. Further studies are necessary to characterize the identified viruses, including virus isolation on mosquito cell lines or genome sequencing, to confirm that these are in fact novel viruses and eliminate the possibility of flavivirus fragments being integrated in the mosquito DNA. 

The ISFV sample for which a larger piece of the NS5 gene region could be amplified was from *An. squamosus* collected in a nature conservation area (Kruger National Park). This particular ISFV was detected in an *Anopheles* species, and therefore constituted a distinct cluster separate from *Aedes* and *Culex* associated ISFV clades [31]. Additionally, Colmant et al. (2017) analyzed a group of *Anopheles* viruses that did not replicate in arthropod cells or heterologous *Anopheles* species, suggesting that these group of viruses could only replicate in their mosquito host, and thus exhibiting an unprecedented specialization for their host species [31]. This may suggest that a better understanding of the different mosquito species that serve as a host could help to elucidate the evolution and ecology of the ISFVs [31]. Mosquito flaviviruses similar to KNP17MP71 were described in *An. gambie* from Liberia and in *An. funestus/An. gambiae/An. rufipes* pools in Senegal [32]; this virus was later detected in *An. gambiae* and *An. squamosus* from Kenya [33]. The data provided in this study are the first to show the presence of an *Anopheles* flavivirus in South Africa and reveal that the sequences are closely related to sequences published from Kenya, with a 96% sequence identity. Further sequence information is required to establish the detailed taxonomic status of the identified virus. 

A cell-fusing agent virus was detected in *Ae. aegypti* and *Ae. caballus* species. This virus was the first mosquito-specific *Flavivirus* identified, isolated and characterized [7]. The first detection was from *Ae. aegypti* cell lines [7], and it was later detected in field-caught *Aedes* and *Culex* mosquitoes in different parts of the world [9,34,35,36]. CFAV appears to be a frequent flavivirus in mosquito populations, and the fact that it was not detected previously in South Africa is mostly likely due to lack of studies on ISFVs as opposed to a lack of presence. The percentage of CFAV-positive pools in *Ae. aegypti* found here (10% of the *Ae. aegypti* pools tested) was similar to a previous study. In Mexico, 10.8% of *Ae. aegypti* mosquitoes tested positive for CFAV Colima strain [34].

Surprisingly, during the period of the study, no ISFVs were detected in *Culex* species and a short fragment of *Culex* flaviviruses-like was identified in two *Anopheles coustani* pools, although it clustered separately to known *Culex flaviviruses*. *Culex* flaviviruses were first reported in Japan and Indonesia and later isolated in different geographical areas such as Brazil [37], Colombia [38], Spain [9], and Uganda [30]. Because of the size of the fragment, is not possible to establish, at this stage, the identification of the virus; however, it appears to be different to the *Culex flaviviruses* and may represent a similar virus adapted to *Anopheles* in Africa. Further studies will be undertaken to characterize this virus and investigate the presence of Culex *flaviviruses* in *Culex* mosquito populations from southern Africa.

The barcode sequencing successfully confirmed most of the morphological mosquito identifications. However, there is a lack of sequences available for African *Culicidae* mosquitoes. This could explain the fact that the sample collected in 2016 from Boschkop (Pretoria) was similar to the *Ae. caballus* sequence from GenBank and later clustered with *Ae. juppi* from South Africa [27]. These two species belong to the *Ochlerotatus* subgenus, are morphologically similar and only a few sequences are available in GenBank for *Ae. juppi*.

## 5. Conclusions

The results obtained in this study suggest that a variety of ISFVs is present in mosquito populations in South Africa. Several different strains were detected, including a cell-fusing agent virus, *Culex* flavivirus-like and *Anopheles* flaviviruses. Further characterization is required to know which virus or strain is in circulation. Although chromosomal DNA was removed as part of the extraction method to rule out the possibility that the viruses identified here were insect-specific DNA integrations in the mosquito genomes, we were only able to amplify larger regions for the four CFAVs and one insect-specific mosquito flavivirus, possibly due to limited conservation in the genome area. Further investigations are needed to characterize the other viruses. These findings confirm the presence of ISFVs in mosquito populations in South Africa, expanding their geographical range and indicating potential mosquito species as vector species.

## Figures and Tables

**Table 1:** *Culicidae* species, vector surveillance site, identification number of Culicidae homogenate pool that tested positive, virus identified, homogenate pool size and NCBI GenBank accession numbers.

*Culicidae* Species	Site	ID Number	Pool Size	Virus	Virus Accession Number	Coi Accession Number
*An. coustani*	KNP	KNP17MP29	39	*Cx.* flavivirus-like	NA < 200 bp	MW077858
*An. coustani*	KNP	KNP17MP30	9	*Cx.* flavivirus-like	NA < 200 bp	MW077859
*An. squamosus*	KNP	KNP17MP71	15	Mosq. Flavivirus	MW655732	NA
*An. maculipalpis*	KNP	KNP17MP673	1	*An.* Flavivirus	NA < 200 bp	NA
*An. squamosus*	KNP	KNP17MP666	1	Mosq. Flavivirus	NA < 200 bp	NA
*An. squamosus*	KNP	KNP17MP667	9	Mosq. Flavivirus	NA < 200 bp	NA
*Ae. aegypti*	Jozini	KZN17MP145	15	Mosq. Flavivirus	NA < 200 bp	MW077851
*Ae. aegypti*	Pretoria	COET17MP500	4	Mosq. Flavivirus	NA < 200 bp	MW077852
*Ae. aegypti*	Pretoria	COET17MP502	21	Mosq. Flavivirus	NA < 200 bp	MW077853
*Aedes* spp	KNP-SHI	SHI17MP544	7	Mosq. Flavivirus	NA < 200 bp	MW077854
*Aedes* spp	KNP	KNP17MP639	4	Mosq. Flavivirus	NA < 200 bp	MW077856
*Aedes vexans* gr.	KNP	KNP17MP653	3	Mosq. Flavivirus	NA < 200 bp	NA
*Ae. sudanensis*	Roodeplat	ROOD18MP126	1	*Ae.* Flavivirus	NA < 200 bp	NA
*Ae. aegypti*	Pretoria	COET17MP503	35	CFAV	MW240714	MW228498
*Ae. aegypti*	Pretoria	COET17MP501	20	CFAV	MW240716	NA
*Ae. caballus*	Boschkop	GAU16MP01	9	CFAV	MW240715	MW077860
*Ae. aegypti*	KNP-SHI	SHI17MP525	50	CFAV	MW240717	MW077855

NA: not applicable, Mosq.: mosquito; *An*.: *Anopheles*, *Ae*.: *Aedes*, *Cx*.: *Culex*, KNP: Kruger National Park, SHI: Shingwedzi, CFAV: cell-fusing agent virus.

## Data Availability

Not applicable.

## Supplementary Materials

The following are available online at https://www.mdpi.com/article/10.3390/v13112148/s1, Table S1: Table with PCR assay, corresponding primers, sequences data, and genome position. Table S2: P-distance matrix showing the percentage (%) nucleotide similarities between mosquito Flavivirus detected in mosquito homogenate pools in the small fragment of the NS5. The highlighted sequences are the sequences produced in this study. Table S3: P-distance matrix showing the percentage (%) nucleotide similarities for the 850 bp region of NS5 between Cell-fusing agent virus detected in mosquito homogenate pools. Table S4: P-distance matrix showing the percentage (%) nucleotide similarities between mosquito Flavivirus detected in mosquito homogenate pools in the large 850 bp fragment of the NS5. Supplementary Material S2: Sequences produced in this study and not deposited at NCBI GenBank.
