# Supplementary material for: Detection of Insect-Specific Flaviviruses in Mosquitoes (Diptera: Culicidae) in Northeastern Regions of South Africa
Source: Viruses. 2021 Oct 25;13(11):2148. doi: 10.3390/v13112148 (PMC8621686; doi:10.3390/v13112148)
Supplement: Supplementary file 1 [file viruses-13-02148-s001.zip › viruses-1391346-supplementary.pdf]

Supplementary Material S1

Table S1: Table with PCR assay, corresponding primers, sequences data, and genome position.

Primer Name	Sequence (5' – 3')	Target gene	Genome position	Ref.
**Flavivirus First round RT-PCR**				
FU1	TACAACATGGGAAAGAGAGAGAA	NS5	9043-9300	(20, 19)
9317	BBGTGATKCKHGTGTCC<br>TCGTGATGCGTGTGTCC	NS5		
**Flavivirus Nested, real time PCR**				
FS778	AARGGHAGYMCDGCHATHTGGT	NS5	9091-9280	(19)
CFD2	GTGTCCCAGCCGGCGGTGTCATCA<br>GC	NS5		
**Flavivirus Heminested PCR**				
MAMD	AACATGATGGGRAARAGRGARAA	NS5	9043	(19)
Flavi-1	AATGTACGCTGATGACACAGCTG<br>GCTGGGACAC	NS5		(1)
Flavi-2	TCCAGACCTTCAGCATGTCTTCTG<br>TTGTCATCCA	NS5		(1)

Table S2: P-distance matrix showing the percentage (%) nucleotide similarities between mosquito *Flavivirus* detected in mosquito homogenate pools in the small fragment of the NS5. The highlighted sequences are the sequences produced in this study.

		1	2	3	4	5	6	7	8	9	10	11	12
1	Kamiti River virus (NC 005064.1)												
2	Nakiwogo virus (GQ165809.2)	67.9											
3	Lammi virus (FJ606789.2)	53.6	44.6										
4	Palm Creek virus (NC033694.1)	62.5	69.6	41.8									
5	Nhumirim virus (KJ210048.1)	50.3	47.3	46.1	47.3								
6	Calbertado virus (KX669687.1)	71.3	62.1	59.6	66.2	45.7							
7	Chaoyang virus (NC 017086.1)	45.1	50.2	71.0	48.4	48.8	49.4						
8	Barkedji virus (KC496020.1)	37.0	32.9	46.4	35.1	74.3	39.4	44.4					
9	Quang Binh virus (FJ644291.1)	71.9	67.3	54.5	70.8	50.1	77.4	50.8	35.5				
10	Culex flavivirus (JX897904.1)	65.7	75.1	47.5	70.3	35.2	70.5	58.5	20.7	73.4			
11	Culex flavivirus (AB262759.2)	60.9	74.1	44.9	70.0	32.9	71.6	51.2	13.1	69.3	93.0		
12	Aedes flavivirus (KC181923)	70.1	63.3	37.7	64.1	36.4	66.6	43.0	23.6	67.3	59.8	63.1	
13	Aedes flavivirus (MK241496.1)	69.8	71.0	50.6	71.0	43.9	61.7	49.8	38.8	65.7	68.1	63.7	68.3
14	Aedes flavivirus Narita-21 (NC 012932.1)	72.3	66.8	36.6	64.1	38.3	67.7	41.2	25.8	69.6	59.5	62.8	97.7
15	Anopheles flavivirus (KX148546.1)	60.8	70.6	46.3	72.2	44.2	65.8	52.3	33.2	67.0	69.1	70.1	59.2

16	Mosquito flavivirus (KY995162.1)	62.3	74.4	57.2	73.3	41.5	69.0	52.5	32.1	74.5	76.6	75.5	61.7
17	Mosquito flavivirus (HQ676625.1)	71.0	66.9	50.4	70.0	38.3	65.0	49.1	33.3	68.0	71.7	68.1	72.1
18	Mosquito flavivirus (HQ676624.1)	71.0	66.9	50.4	70.0	38.3	65.0	49.1	33.3	68.0	71.7	68.1	72.1
19	Culiseta flavivirus (KT599442.1)	71.7	68.3	53.1	71.7	37.8	73.1	48.5	28.3	74.1	74.9	69.6	70.3
20	KNP17MP30 An. coustani	60.8	65.5	45.2	67.2	34.3	70.7	47.6	26.6	67.3	77.2	76.2	61.0
21	KNP17MP29 An. coustani	54.1	61.5	36.5	58.4	24.7	62.3	46.2	16.5	59.9	73.8	72.8	54.1
22	KNP17MP667 An. squamosus	59.6	62.0	42.6	76.5	28.5	66.9	42.0	29.2	69.0	65.9	60.9	60.0
23	KNP17MP71 An. squamosus	45.2	52.1	33.6	43.8	15.6	49.3	39.3	6.3	52.5	68.0	73.6	51.3
24	KNP17MP666 An. squamosus	57.4	64.1	41.7	73.6	30.0	64.8	37.6	29.7	69.0	65.9	60.9	60.2
25	KNP17MP673 An. maculipalpis	61.6	67.0	51.6	71.4	43.0	64.7	57.7	32.1	70.3	69.1	64.8	63.2
26	COET17MP500 Ae. aegypti	66.2	63.1	43.6	66.4	30.8	61.1	42.4	25.2	64.3	68.1	64.2	67.4
27	COET17MP502 Ae. aegypti	71.0	66.9	50.4	70.0	38.3	65.0	49.1	33.3	68.0	71.7	68.1	72.1
28	KZN17MP145 Ae. aegypti	70.0	72.0	47.7	70.0	43.9	60.6	47.0	38.8	65.8	70.3	66.0	68.3
29	ROOD18MP126 Ae. sudanensis	64.1	65.9	48.6	62.5	39.6	56.5	44.5	37.7	59.7	62.6	57.9	60.2
30	KNP17MP653 Ae. vexans gr	57.4	64.1	41.7	73.6	30.0	64.8	37.6	29.7	69.0	65.9	60.9	60.2
31	SHI17MP544 Aedes spp.	69.8	71.0	50.6	71.0	43.9	61.7	49.8	38.8	65.7	68.1	63.7	68.3
32	KNP17MP639 Aedes spp.	62.9	66.8	52.9	71.5	54.0	70.2	54.4	35.0	79.4	68.5	65.8	67.8

		8	9	10	11	12	13	14	15	16	17	18	19
13	Aedes flavivirus (MK241496.1)												
14	Aedes flavivirus Narita-21 (NC 012932.1)						68.5						
15	Anopheles flavivirus (KX148546.1)						62.6	58.1					
16	Mosquito flavivirus (KY995162.1)						68.7	62.8	73.0				
17	Mosquito flavivirus (HQ676625.1)						91.3	72.1	62.7	68.8			
18	Mosquito flavivirus (HQ676624.1)						91.3	72.1	62.7	68.8	100.0		
19	Culiseta flavivirus (KT599442.1)						70.7	67.9	64.2	66.9	72.0	72.0	
20	KNP17MP30 An. coustani						62.9	60.8	67.2	68.9	74.6	74.6	73.7
21	KNP17MP29 An. coustani						52.0	53.8	59.8	61.3	65.3	65.3	66.8
22	KNP17MP667 An. squamosus						72.3	61.3	71.9	59.3	79.3	79.3	65.0
23	KNP17MP71 An. squamosus						44.8	50.9	40.3	52.0	51.8	51.8	52.2
24	KNP17MP666 An. squamosus						71.4	61.6	68.9	59.3	78.5	78.5	61.6
25	KNP17MP673 An. maculipalpis						66.0	63.3	89.6	69.7	69.5	69.5	62.0
26	COET17MP500 Ae. aegypti						87.8	67.3	58.6	65.1	96.9	96.9	68.5
27	COET17MP502 Ae. aegypti						91.3	72.1	62.7	68.8	100.0	100.0	72.0
28	KZN17MP145 Ae. aegypti						98.5	68.5	61.5	70.8	91.3	91.3	69.7
29	ROOD18MP126 Ae. sudanensis						93.7	60.5	53.0	61.7	84.9	84.9	64.9
30	KNP17MP653 Ae. vexans gr						71.4	61.6	68.9	59.3	78.5	78.5	61.6
31	SHI17MP544 Aedes spp.						100.0	68.5	62.6	68.7	91.3	91.3	70.7
32	KNP17MP639						72.4	68.9	67.9	70.2	69.3	69.3	72.2

	Aedes spp.

Table S3: P-distance matrix showing the percentage (%) nucleotide similarities between Cell fusing agent virus detected in mosquito homogenate pools. The highlighted sequences are the sequences produced in this study.

		1	2	3	4	5	6	7
1	KJ741267.1 CFAV Galveston							
2	LR694075.1 CFAV Cairns	97.03%						
3	HM579916.1 CFAV Colima	96.21%	95.99%					
4	KP792624.1 CFAV Kenya	95.81%	96.01%	94.55%				
5	COET17MP503 Ae. aegypti	94.58%	95.58%	93.70%	95.61%			
6	COET17MP501 Ae. aegypti	93.55%	94.55%	93.08%	95.40%	97.22%		
7	GAU16MP01 Ae. caballus	93.97%	94.97%	93.51%	95.41%	97.42%	98.03%	
8	SHI17MP525 Ae. aegypti	93.54%	94.55%	92.65%	95.00%	98.02%	95.79%	96.61%

Table S4: P-distance matrix showing the percentage (%) nucleotide similarities between mosquito *Flavivirus* detected in mosquito homogenate pools in the large fragment of the NS5. The highlighted sequence is the sequence produced in this study.

		1	2	3	4	5
1	Aedes flavivirus (KC181923)					
2	Aedes flavivirus Narita-21 (NC 012932.1)	98.24%				
3	Mosquito flavivirus (KM088035.1)	40.12%	40.65%			
4	Mosquito flavivirus (KM088039.1)	40.08%	40.62%	99.73%		
5	Haslams Creek (MF352617.1)	38.84%	39.95%	72.89%	73.28%	
6	KNP17MP71 An. squamosus	37.57%	38.11%	96.58%	96.86%	73.02%

Supplementary Material S2: Sequences produced in this study and not deposited at NCBI GenBank.

>KNP17MP30 An. coustani

TGGCTGGGAAGTCGATTTCTCGAGTACGAAGCACTAGGATTCCTGAATG
CGGACCATTGGGTAGCTAGAATGAATTTACCCTGTGGGGTCGGAGGAGTTGGG
GTGAACTACTTTGGTAATTATCTCAAGGAGAT

>KNP17MP29 An. coustani

TGGCTAGGAAGTCCGATTCTCGAGTACGAAGTACTAGGATTCCTGAATG
CGGACCATTGGGTAGCTAGAATGAATTGAACCTGCGGGGTCGGAGGAGTTGGG
GTGAACTACTTTGGTAATTATCTCAAGGAGAT

>KNP17MP667 An. squamosus

TGGTTAGGAAGTCGATATCTAGAGTATGAGGCTTTAGGTTTCCTCAACC
AAGACCATTGGGTTTCACGTGACCATCTGCCATGTGGAGTGGGAGGCGTTGGT
GTGAACTATTTTGGTAATTATCTGAAGGAAAT

>KNP17MP71 An. squamosus

TGGCTCGGAAGTCGGTTTTTGGAGTACGAAGCACTTGGGTTCCTAAACG
AGGATCACGGTTTAACCAGCAAAAATATTGCCGACAGGGTTGCCAGATCTCGC
TTGCATTACTTTGGATACTATCTCCAGGAGAT

>KNP17MP666 An. squamosus

TGGTTAGGAAGTCGATATCTAGAGTATGAGGCTTTGGGTTTTCTCAACC
AAGACCATTGGGTTTCACGTGACCATCTGCCATGCGGAGTGGGAGGCGTTGGT
GTGAACTATTTTGGTAATTATCTGAAGGAAAT

>KNP17MP673 An. maculipalpis

TGGCTAGGGAGTAGATTCTTGGAATATGAAGCCCTAGGATTCTTAAACC
AAGACCATTGGGTGTCACGTGACCATTTGCCATGTGGGGTCGGAGGCGTTGGT
GTCAATTACTTCGGAAATTACCTGAAGGAAAT

>COET17MP500 Ae. aegypti

TGGCTGGGAAGTCGATATCTGGAATATGAGCCCTTGGGCTTCCTCACCG
AAGACCATTGGGTAGCCCGTGAAAATTTCCCGAATGGCGTGGGAGGTCTCGGT
GTGAACTACTTTGGTAATTATCTCAAGGAGAT

>COET17MP502 Ae. aegypti

TGGCTGGGAAGTCGATATCTGGAATATGAGGCCTTGGGCTTCCTCAACG
AAGACCATTGGGTAGCCCGTGAAAATTTCCCGGGTGGCGTGGGAGGTCTCGGT
GTGAACTACTTTGGTAATTATCTCAAGGAGAT

>KZN17MP145 Ae. aegypti

TGGCTGGGAAGTCGATATCTGGAATATGAGGCCTTGGGCTTCCTCAACG
AAGACCATTGGGTAGCCCGTGAAAATTTCCCGGGCGGCGTGGGAGGTCTCGGC
GTGAACTACTTTGGCTATTACTTGTCAGAAAT

>ROOD18MP126 Ae. sudanensis

TGGCTGGGAAGTCGATATCTTGGATATGAGGCCTTGGGCTTCCTCAACG
AAGACCATTGGGTAGCCCGTGAAAATTTCCCGGGCGGCGTGGGAGGTCTCGTA
CTATACTACTTTGGCTATTACTTGTCATAAAT

>KNP17MP653 Ae. vexans gr

TGGTTAGGAAGTCGATATCTAGAGTATGAGGCTTTGGGTTTTCTCAACC
AAGACCATTGGGTTTCACGTGACCATCTGCCATGCGGAGTGGGAGGCGTTGGT
GTGAACTATTTTGGTAATTATCTGAAGGAAAT

>SHI17MP544 Aedes spp.

TGGCTGGGAAGTCGATATCTGGAATATGAGGCCTTGGGCTTCCTCAACG
AAGACCATTGGGTAGCCCGTGAAAATTTCCCGGGTGGCGTGGGAGGTCTCGGC
GTAAACTACTTTGGCTATTACTTGTCAGAAAT

>KNP17MP639 Aedes spp.

TGGTTGGGCAGTAGGTTCTTGGAGTATGAGGCCCTGGGGTTTTTAAATGAGGAT
CACTGGGTAGCACGGGAGAATTTCCCTGGTGGTGTCGGAGGCATGGGCGTTAA
CTACTTTGGCTATTACCTGGAGGAAAT
